# Elevation of dopamine level reduces host-seeking activity in the adult female mosquito *Aedes albopictus*

**DOI:** 10.1186/1756-3305-5-92

**Published:** 2012-05-10

**Authors:** Yuki Fukumitsu, Keiichi Irie, Tomomitsu Satho, Hitoshi Aonuma, Hamady Dieng, Abu Hassan Ahmad, Yukihiko Nakashima, Kenichi Mishima, Nobuhiro Kashige, Fumio Miake

**Affiliations:** 1 Faculty of Pharmaceutical Sciences, Fukuoka University, 8-19-1 Nanakuma, Jonan-ku, Fukuoka, 814-0180, Japan; 2 Research Institute for Electronic Science, Hokkaido University, Sapporo, Hokkaido, 060-0812, Japan; 3 School of Biological Sciences, Universiti Sains Malaysia, Penang, 11800, Malaysia

**Keywords:** Host-seeking behavior, Dopamine, *Aedes albopictus*

## Abstract

**Background:**

Mosquito-borne viruses are transmitted to human hosts *via* blood-feeding behavior of female mosquitoes. Female mosquitoes seek a host to take blood meals (host-seeking behavior). In order to prevent virus infections, it is important to understand how they modulate host-seeking behavior. Dopamine (DA) in the central nervous system acts as a neuromediator that regulates a variety of behaviors in insects. In female mosquitoes, host-seeking behavior increases when DA levels in the head decline after emergence. However, it remains unclear whether DA directly modulates host-seeking behavior in female mosquitoes. The aim of this study was to examine whether changes in DA levels in the head affects host-seeking activity in the adult female mosquito *Aedes albopictus* (*Ae. albopictus*).

**Findings:**

We compared host-seeking behavior in one group of emerging female adults treated with l-β-3,4-dihydroxyphenylalanine (l-DOPA), the precursor of DA, (l-DOPA group), with that in an untreated control (control group) after confirming elevation of head DA in l-DOPA group by using high-performance liquid chromatography. The content of head DA in l-DOPA group significantly remained higher than that in controls on all days examined. The host-seeking activity in the control group showed a gradual increase over the 6-day experimental period. In contrast, there was no such increase in the host-seeking activity in the l-DOPA group. Therefore, the host-seeking activity of l-DOPA group was significantly lower than that of the controls between day 3 and 6 post-emergence.

**Conclusion:**

Our results indicate that elevation of DA level reduces host-seeking activity in adult female mosquito *Ae. albopictus*.

## Findings

*Aedes albopictus* (*Ae. albopictus*) is known as a vector of mosquito-borne viruses (e.g. Dengue virus and West Nile virus) [1]. Female mosquitos seek a host to take blood meals (host-seeking behavior). In order to prevent virus infections, it is important to understand how they modulate host-seeking behavior.

In the central nervous system of insects, biogenic amine dopamine (DA) acts as a neuromediator (*i.e.* neurotransmitter, neuromodulator and neurohormone) to modulate a wide variety of behaviors, including insemination, diapause, and locomotory behavior [2-4]. In mosquitoes, it is demonstrated that DA levels in the head gradually decrease day by day after adult emergence [5]. Host-seeking behavior, on the other hand, is shown to increase after adult emergence. Interestingly, the period of change in DA level coincides with that in which host-seeking behavior changes. These observations suggested that DA could play a role in modulating host-seeking behavior in the mosquito. In female mosquitoes, however, the direct relation between DA in the head and host-seeking behavior remained unclear. In order to elucidate the role of DA in host-seeking behavior, we examined whether change in DA level in the head affects host-seeking activity in the adult female mosquito *Ae. albopictus*. The present study strongly suggests that elevation of DA level in the head inhibits host-seeking behavior of adult female mosquito *Ae. albopictus*.

*Ae. albopictus* mosquitoes used in this study were from a laboratory colony established from adults collected in Fukuoka university. All mosquitoes were reared in our laboratory on a 16h:8h light and dark cycle at 27 ± 2°C and 70 ± 10% RH as described previously [6]. Female and male pupae were collected into different chambers respectively. Adult mosquitoes emerging on the same day were moved into a cage (200 individuals with a sex ratio of 1:1). For elevation of DA level in heads of insects, l-β-3,4-dihydroxyphenylalanine (l-DOPA), the precursor of DA, is used as described previously [3,7]. The cages were separated into two groups: one receiving 3% sucrose solution containing 0.07% l-DOPA (l-DOPA group) and another receiving only 3% sucrose solution (control group).

High-performance liquid chromatography (HPLC) with electrochemical detection (ECD) was used to measure the DA levels in the heads of mosquitoes as described previously [8]. The heads of mosquitoes were separated from the body laid on ice under a stereomicroscope [5].

The heads of adult female mosquitoes were collected on days 0, 3, and 6 after emergence. A single head of mosquito was sufficient to achieve a robust DA signal by using HPLC-ECD.

**Figure 1:**
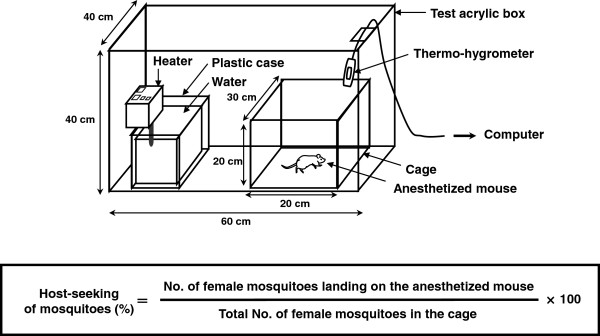
**Schematic view of the apparatus used and equation to evaluate host-seeking activity.** A test acrylic box was maintained under the same conditions as used for rearing. Mosquitoes landing on the mouse were judged to show host-seeking behavior and number of them was counted.

Host-seeking activity of mosquitoes was evaluated once a day for 6 days after emergence. Behavior experiments were performed 7–8 h after the dark cycle when more mosquitoes were likely to show host-seeking behavior [9]. To allow the mosquitoes to acclimatise to the experimental environment, the mosquitoes in the cage were kept in a test acrylic box for 5 min before the tests (Figure 1). The condition inside the test box was the same as the rearing conditions.

An anesthetized mouse (BALB/c: 5–7 weeks old, Kyudo Co., Saga, Japan) was placed in the cage in the test box and used as a host. Then host-seeking behavior of female mosquito was observed for 30 min. Mosquitoes that succeeded in landing on the mouse were judged as showing host-seeking behavior and the number observed landing was counted. We then collected these mosquitoes quickly using a mouth aspirator before they started blood feeding.

The mouse was then removed from the cage and the collected mosquitoes were returned to the same cage for assessment on the following day. Host-seeking activity was evaluated by determining the percentage as (No. of female mosquitoes landing on the anesthetized mouse in 30 min/Total No. of female mosquitoes in the cage, Figure 1). All procedures regarding animal care and use were performed in compliance with the regulations established by the Experimental Animal Care and Use Committee of Fukuoka University. Behavior experiments were performed under pentobarbital anesthesia, and all efforts were to minimize suffering.

The results are expressed as the means ± standard error of the mean (S.E.M.). To test for significance between treatments and for interaction between treatments and collection days in DA level, two-way ANOVA was applied followed by Student’s *t*-test. Two-way repeated measures ANOVA was performed to test for significance between treatments and for interaction between treatments and collection days in host-seeking activity, and differences between control and l-DOPA groups were tested by Tukey’s test. In all analyses, *P* < 0.05 was taken to indicate statistical significance.

**Figure 2:**
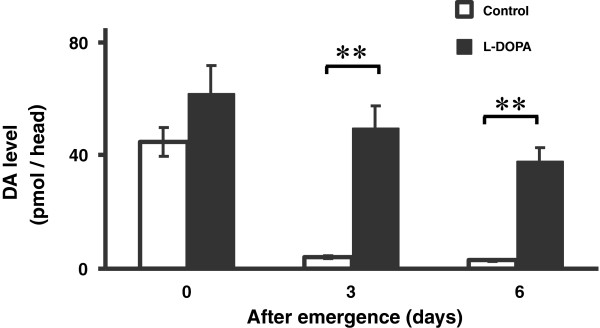
**DA levels in the head on days 0, 3, and 6 after emergence.** ** *P* < 0.01 vs. control group (two-way ANOVA followed by Student’s *t*-test). Data are means ± S.E.M., *n* = 20 samples per treatment.

**Figure 3:**
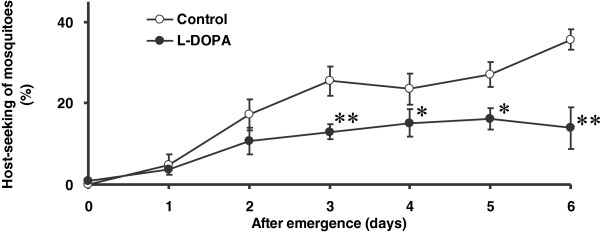
**Host-seeking activity during the six days after emergence**. * *P* < 0.05, ** *P* < 0.01 vs. control group (two-way repeated measures ANOVA followed by Tukey’s test). Data are means ± S.E.M., *n* = 6 samples per treatment.

In the l-DOPA group receiving a sucrose solution containing 0.07% l-DOPA, we found that the DA levels in the head remained significantly higher than those in controls on days 3 and 6 post-emergence (control group, day 0: 44.6 ± 5.0; day 3: 4.1 ± 0.3; day 6: 2.9 ± 0.4 pmol/head; l-DOPA group, day 0: 62.3 ± 9.3; day 3: 49.7 ± 7.7; day 6: 38.0 ± 5.0 pmol/head; Group, F_1, 114_ = 49.28, *P* < 0.01; Day, F_2, 114_ = 18.75, *P* < 0.01; Group×Day, F_2, 114_ = 3.04, *P* > 0.05: two-way ANOVA, Figure 2). The host-seeking activity showed a gradual increase over the 6-day experimental period in the control group (day 0: 0.0 ± 0.0%; day 1: 4.9 ± 2.6%; day 2: 17.2 ± 3.9%; day 3: 25.5 ± 3.6%; day 4: 23.5 ± 3.9%; day 5: 27.1 ± 3.1%; day 6: 35.7 ± 2.6%). In contrast, there was no such increase in host-seeking activity in the l-DOPA group (day 0: 0.8 ± 0.4%; day 1: 3.7 ± 1.4%; day 2: 10.7 ± 3.3%; day 3: 13.0 ± 1.8%; day 4: 15.1 ± 3.4%; day 5: 16.1 ± 2.6%; day 6: 13.9 ± 5.1%). Therefore, the host-seeking activity of mosquitoes in the l-DOPA group was significantly lower than that in the control group between days 3 and 6 post-emergence (Group, F_1, 10_ = 24.33, *P* < 0.01; Day, F_6, 60_ = 19.16, *P* < 0.01; Group×Day, F_6, 60_ = 3.18, *P* < 0.01: two-way repeated measures ANOVA, Figure 3).

In female mosquitoes, the direct relationship between DA in the head and host-seeking behavior remains unclear. Our results demonstrated that an increase in DA level using l-DOPA decrease host-seeking behavior between days 3 and 6 post-emergence. We describe here the first evidence that DA in the head of the adult female mosquitoes of *Ae. albopictus* is involved in modulating host-seeking behavior because elevation of DA levels in the head decreases host-seeking behavior.

Up to now, biogenic amine has received attention for regulating the behavior of insects. In the previous study, pharmacological treatment using amine depleting drugs α-methyl-tyrosine (AMT) and α-methyl-tryptophan (AMTP), demonstrated that depleted DA levels in the mosquito *Aedes triseriatus* that were 3 day old adult females did not affect host-seeking behavior, however, serotonin (5HT) depletion modulated blood-feeding behavior [10]. Our study could suggest the reason why depleted DA levels did not affect host-seeking behavior in the previous study. In Figure 2, our results demonstrated that head DA levels in 3 day old females in control mosquitoes decreased sufficiently. Therefore, it might be difficult to observe an effect of depleted DA level on host-seeking behavior in the previous study. Our study also demonstrates that manipulation of DA as well as 5HT makes it possible to regulate the behavior of mosquitoes.

In insects, the presence of DA neurons has been demonstrated [4,7,8]. In a previous study, adult *Drosophila melanogaster* (*Drosophila*) were treated with a solution of sucrose containing l-DOPA in order to increase the DA level in the central nervous system in the head [7]. In our study, adult mosquitoes were also given a sucrose solution containing 0.07% l-DOPA, and our results indicated that the DA level in the head in the l-DOPA group was significantly higher than that in controls. Thus, feeding with a sucrose solution containing l-DOPA is an appropriate method to increase the level of DA in the head of not only *Drosophila* but also *Ae. albopictus* mosquitoes.

Our results confirmed that the DA level in the head in the control group decreased after adult emergence, consistent with findings reported previously [5], and that the host-seeking activity increased gradually over the experimental period in the control group (Figures 2 and 3). These observations indicated the reliability of our methods for quantification of DA levels in the head and host-seeking behavior.

Further investigation to elucidate the role of biogenic amine DA in the mechanism of host-seeking behavior regulation of the mosquito must contribute to the prevention of epidemics of mosquito-borne viruses.

## Competing interests

The authors declare that they have no competing interests.

## Authors’ contributions

Conceived the idea: YF, KI. Performed the experiment and analyzed the data: YF, KI. Contributed reagents/materials/analysis tools: KI, TS, HA and YN. Wrote the manuscript: YF. Clarified the manuscript: KI, TS, HA, HD, AH, KM, NK, and FM. All authors read and approved the final manuscript.
